# Use of Global initiative for asthma (GINA) guidelines in asthma management among paediatric residents in a Sub Saharan African country: a cross-sectional descriptive study

**DOI:** 10.11604/pamj.2017.27.120.9260

**Published:** 2017-06-15

**Authors:** Adaeze Chikaodinaka Ayuk, Agozie Ubesie, Chioma Laura Odimegwu, Kenechukwu Iloh

**Affiliations:** 1 Department of Paediatrics, University of Nigeria Teaching Hospital, Enugu, Nigeria; 2 College of Medicine, University of Nigeria Enugu Campus, Nigeria

**Keywords:** Asthma, children, Paediatric residents, GINA guidelines, Nigeria

## Abstract

**Introduction:**

Clinical practice guidelines are systematically developed statements to assist practitioner and patient decisions about appropriate health care for specific clinical circumstances. Despite abundance of asthma guidelines, prevalence has continued to increase globally. There is need to assess how the contents of asthma guidelines are put to clinical use by doctors in the management of children with asthma. This study aims at evaluating the clinical practice of paediatric residents in applying GINA guidelines.

**Methods:**

Cross-sectional descriptive study of paediatric residents from 23 university teaching hospitals in Nigeria using structured questionnaire. Data analyses were with Statistical Package for Social Sciences (SPSS) version 19 (Chicago IL). Chi square was used to assess for any significant associations between categorical variables. A p < 0.05 was regarded to be statistically significant.

**Results:**

Sixty-six paediatric residents aged 27- 40 years were enrolled into the study (37 females and 29 males). One-third had spent more than three years in residency training. Fifty-eight residents (87.9%) were aware of the GINA guidelines while 46 (69.7%) were familiar with its contents. Only 39 (59.1%) residents adhered to the GINA guidelines. Twenty of the 35 junior residents (57.1%) compared to 26 of 31 (83.9%) senior residents were familiar with the GINA guidelines (p=0.031) while 15 of 35 junior residents (42.9%) compared to 24 of 31 senior residents (77.4%) consistently follow the GINA guidelines (p=0.006). Adherence to GINA guidelines was not influenced significantly by years of graduation or training (p>0.05).

**Conclusion:**

The use of the GINA guidelines was poor among paediatric residents. Application of contents rather than just availability of asthma guidelines may partly account for increasing asthma prevalence globally.

## Introduction

The control of asthma especially in children has continued to be a challenge and non-adherence to guidelines for asthma management by practitioners is noted as a cause [1, 2]. Clinical practice guidelines are systematically developed statements to assist practitioner and patient decisions about appropriate health care for specific clinical circumstances. Use of asthma guidelines by physicians who care for children could reduce paediatric emergency department visits and hospitalizations thus saving an estimated $1.3 billion annually [2]. Despite the availability of guidelines, asthma burden and prevalence have not improved significantly [3]. The lack of published literature on the in-depth study of how these guidelines have been applied by those who should be using it may reveal where the true gaps are. There are several asthma guidelines, which are usually adapted to specific country needs. Nigeria as a country as at the time of this research, had no National Asthma Management Guideline. This is despite the fact that 15-20 million Nigerians are either suffering from asthma or are having symptoms suggestive of asthma [4]. In the absence of a locally adapted guideline, the practice of asthma management in Nigeria, until recently, was generally based on adaptations from Global Initiative for Asthma management (GINA) guidelines. The Global Initiative for Asthma (GINA) was established to increase awareness about asthma among health professionals, public health authorities and the community, and to improve prevention and management through a coordinated worldwide effort. GINA prepares scientific reports on asthma, encourages dissemination and implementation of the recommendations, and promotes international collaboration on asthma research1The practicability of some of the recommendations may be a challenge especially in resource poor countries where information dissemination and availability of standard medications may not be as expected. The paediatric residents in training within the teaching hospitals form a major bulk of the work force involved in the management of Nigerian children with asthma and thus a representative population of asthma management in tertiary hospitals in Nigeria. There are not many published studies that have critically analysed guideline content use. There is need to assess how much of the contents of GINA guidelines are put to clinical use by doctors as this will impact on asthma outcomes. This will help inform policy makers both locally and globally on how best to adapt GINA guidelines to country-specific needs so as to improve asthma outcomes. The aim of this study was to critically analyse guideline content use by paediatric residents in a country without its own National asthma guideline.

## Methods

This was a cross-sectional descriptive study of resident doctors in paediatrics participating in a two- week annual National postgraduate Research and Update Course, held in August 2015 in Lagos Nigeria. The residents were drawn from 23 paediatric residency training centers in Nigeria. One hundred and one paediatric residents participated in the National workshop. Among them ninety-nine consented to the study and 72 returned their completed questionnaires. All consenting paediatric residents from these teaching hospitals attending this national event were sampled consecutively (convenient sampling). A structured pre-tested questionnaire designed by the paediatric asthma consortium written in English was self-administered. It was returned same day to the researchers, thus eliminating the need to contact participants for questionnaire return and further narrowing non-response rate. The questionnaire was pre-tested to ensure internal consistency and validity before use. The questions were structured to fit GINA guideline on diagnostic criteria for asthma, assessment of asthma, treating to control symptoms and minimize future risk, management of worsening asthma and exacerbations in adults, adolescents, and children 6 to 11 years. Aspects of questionnaire that reported on how the responding doctors practiced these GINA recommendations made up of 20 multi-structured questions on diagnosis, patient assessment, drug treatment, home monitoring and follow-up of paediatric asthma patients, were assessed. Information on years of practice and exposure to any asthma CME courses was also collected. A total of 101 questionnaires were distributed and 72 were returned giving a response rate of 71.3%. Six of the questionnaires had several missing data and were excluded from the final analyses. The participants were made up of junior residents of less than three years in postgraduate training and senior residents of three or more years in practice. Prior to commencing the study ethical approval was obtained from the Human Research and Ethics Committee of the University of Nigeria Teaching Hospital Enugu.

**Statistical analyses**: Data analyses were with Statistical Package for Social Sciences (SPSS) version 19 (Chicago IL). Questions with multiple answers were transformed to binary answers (yes/no). Other aspects on knowledge of GINA contents were analysed separately. Percentages of doctors who engaged in different aspects of practice were calculated. Practice points with low percentages were further analysed to assess possible predictors of poor practice of asthma management guidelines and reasons proffered for low adherence among these doctors. Chi square was used to check for any significant associations between categorical variables. A p < 0.05 was regarded to be statistically significant. All reported p-values were two sided.

## Results

### General data

Sixty-six paediatric residents aged 27 to 40 years were enrolled into the study. Forty-one of the 66 residents (62.1%) were aged 31 to 35 years. Thirty-seven (56.1%) were females and 35 (53.0%) junior residents. Thirty-five (53%) graduated six to 10 years ago from medical school. An approximate one-third has spent more than three years in residency training. The details are shown in Table 1. Fifty-eight residents (87.9%) were aware of the GINA guidelines, 46 (69.7%) reported being familiar with contents of the GINA guidelines while 39 (59.1%) follow the GINA guidelines in their management of paediatric asthma. Table 2 shows the proportion of residents that were familiar with the practice contents of GINA and their level of compliance.

**Table 1:** Demographic characteristics of all study participants

Categories	n (%)	
Age group (years)	27-30	18 (27.3)
	31-35	41 (62.1)
	36-40	7 (10.6)
Gender	Male	29 (43.9)
	Female	37 (56.1)
Stage of residency	Junior	35 (53.0)
	Senior	31 (47.0)
Post-graduation duration (years)	0-5	27 (40.9)
	6-10	35 (53.0)
Duration of residency training (years)	>10	4 (6.1)
	≤ 1	13 (19.7)
	1.1-2	19 (28.8)
	2.1-3	11 (16.7)
	>3	23 (34.8)

p>0.05

**Table 2:** Performance of all study participants on clinical practice of asthma management

Clinical practice parameters	Yes (%)	No (%)
Confirms asthma diagnosis with pulmonary functions tests	31 (47.0)	35 (53.0)
Familiar with peak flow meter	48 (72.7)	18 (27.3)
Familiar with spirometer	12 (18.2)	54 (81.8)
Assesses inhaler technique at each visit	35 (53.0)	31 (47.0)
Assesses treatment adherence at each visit	56 (84.8)	10 (15.2)
Identify triggers and attempt at environmental manipulations	57 (86.4)	9 (13.6)
Uses guideline to assess asthma control	35 (53.0)	31 (47.0)
Addresses patients’ concerns	57 (86.4)	10 (15.2)
Insists on spacer with face mask for children <6years	36 (54.5)	30 (45.5)
Routinely provides written asthma action plan	24 (36.4)	42 (63.6)
Prescribes inhaled corticosteroids as initial maintenance therapy	12 (18.2)	54 (81.8)
Prescribes LTRA when ICS only is not available for children	17 (25.8)	49 (74.2)
Prescribes ICS/LABA combination	23 (34.8%)	43 (65.2)
Checks allergy status of patients	12 (18.2)	54 (81.8)
Allows 3 months on current medications before stepping up	31 (47.0)	35 (53.0)
Refers to asthma nurse counsellors	21 (31.8)	45 (68.2)

### Specific practice points

***Asthma diagnosis***: To assess how the doctors made a definitive diagnosis of asthma on first clinic visit beyond clinical history, especially among older children, they were asked if they were familiar with use of spirometer machine or peak flow meter and if they confirmed asthma diagnosis by airflow limitation tests. Only 12 residents (18.2%) were familiar with the spirometer machine while 31(47%) performed any lung function tests as part of diagnosis (Table 2).

***Further assessment of patient***: to assess how asthma control is monitored, the doctors were asked if they assessed the children’s inhaler technique at each visit and if adherence to prescribed treatment were assessed at each visit. Attempt at helping the patient identify and control environmental triggers and addressing of patients concerns and use of GINA guidelines to assess asthma control were also evaluated as shown in Table 2. Thirty-five (53%) of respondents used the guideline to assess asthma control.

***Medication prescription***: To assess if the guideline recommendations influenced how prescription for asthma treatment was done, question on drug prescription were asked. Twelve of the 66 residents (18.2%) prescribed inhaled corticosteroids (ICS) as initial maintenance treatment, 17 (25.8%) prescribed leukotriene receptor antagonist (LTRA) when ICS only formulation is not available while 23 (34.8%) prescribed inhaled CS with a long acting B agonist (LABA) combination. Also the use of spacer (with a face mask for children less than 6 years) in administering pressurized metered dose inhalational drugs was also assessed. This showed that 36 (54.5%) of the paediatric residents engaged in this practice.

***Home monitoring***: To find out if doctors wrote down for patients an asthma action plan to take home especially for patients that own a personal peak flow meter, we noted that about a third (36.4%) of the residents routinely provide asthma action plan (36.4%) as shown in Table 2.

***Follow-up visits***: Questions included use of Asthma control Test (ACT) to assess level of control, routine check of allergy status of patients, use of a current prescription for about 3 months before stepping up or stepping down (step ladder approach), referral to asthma educator/counsellor for further asthma education re-enforcement of technique, referring to a specialist because asthma is difficult to control or diagnosis is uncertain, scheduling patients for regular check-up. Only 12 residents (18.2%) did check allergy status of their patients and about a third (31.8%) of the residents referred clients to asthma counsellors. Of those who did refer, 18 of 37 females (48.6%) compared to three of 29 males (10.3%) routinely refer clients to asthma counsellors (p=0.001).

#### Residents’ performance in practice of GINA management guidelines

A further breakdown showed that 20 of the 35 junior residents (57.1%) compared to 26 of 31 (83.9%) senior residents were familiar with the GINA guidelines (p=0.017) (Table 3). Similarly, 15 of 35 junior residents (42.9%) compared to 24 of 31 senior residents (77.4%) follow the GINA guidelines (p=0.006). Forty of the 66 residents (60.6%) attributed non-adherence to the GINA guidelines to irregular asthma CMEs and trainings while 5 (7.6%) and 21 (31.8%) residents attributed it to lack of basic asthma management equipment and insufficient exposure to respirology rotation. Forty-one of the 66 residents (62.1%) had attended Continuing Medical Education (CME) seminar or workshop on asthma since graduation. Half of the respondents had an asthma training more than five years ago while nine (13.6%) and 24 (36.4%) had their last asthma training within 1 to 5 years and less than one year ago respectively. Adherence to GINA guidelines was not influenced significantly by years of graduation, stage of residency training or, exposure to asthma CME (p>0.05).

**Table 3:** GINA guideline use for asthma management by cadre of paediatric resident

	Junior residents (n= 35)	Senior residents (n= 31)	p value
Familiar with the GINA guidelines	20 (57.1%)	26 (83.9%)	0.017
Follow the GINA guidelines	15 (42.9%)	24 (77.4%)	0.006

## Discussion

Our study showed that very few paediatric residents were either familiar with tools used in diagnosis and assessment of asthma or even employed them in their daily practice. Even though asthma diagnosis can be made with history of symptoms and signs, particularly in children, proper classification and follow-up requires the use of physiological assessment of lung function. Spirometry is the gold standard for making a definitive asthma diagnosis [1, 5]. Peak flow meters though more common, are more useful for monitoring progress in already diagnosed individuals [5, 6]. In the presence of numerous asthma mimics, over treatment would be an issue if correct diagnosis is not made. Inadequate medication use would also be a problem. This would contribute to high prevalence of poorly controlled asthma. In a South African study, only 23.2% of physicians were performing peak expiratory flow (PEF) measurements in their patients [7]. Ayuk et al [8], reported that only 34% of doctors in three south-eastern states of Nigeria used lung function measures to make a diagnosis of asthma. Another Nigerian study [9], showed that only 38% and 29.4% of University Teaching hospitals possessed peak flow meter or spirometers respectively. The unavailability of equipment may be a major deterrent to application of GINA guidelines in proper asthma diagnosis/management among paediatric residents managing children with asthma in Nigeria. The GINA guidelines emphasize that inhaler technique and adherence be assessed at each visit. Only about half of our study population carried these out. A possible explanation could be because many of the residents had not received additional medical education courses on asthma management since graduation. However, Mash et al [7], in South Africa equally noted that only 14% of patients had their inhaler technique assessed by doctors. This further suggests that beyond formal trainings, there may be need to enforce the use of guidelines even in countries where the resources are available. Paediatric residents in our study population were noted to be poor in assessing asthma control. This was comparable to the South African study, where only 11.5% of the visits to the doctors recorded having assessed control [7]. This has negative impact on correct asthma status classification of the patient and thus correct use of asthma medications. Guideline recommendations include use of appropriate medications and for appropriate treatment period.

Inhaled corticosteroid (ICS) is the preferred mainstay of treatment for persistent asthma symptoms. Very few of the residents, prescribed ICS as initial maintenance treatment. A few more understood the GINA step 2 where LTRA could be an alternative to ICS especially in the very young children. More respondents actually did prescribe inhaled CS with a long acting B agonist combination. The choices may not be only due to lack of knowledge of specific GINA recommendations, but attributable to availability and cost of the recommended drugs. The LABA combinations are the only easily available local option of ICS in the Nigeria even for paediatric practice despite safety concerns for use of LABA in children [10]. ICS alone medications are not readily available even though related pharmaceutical companies in Nigeria are aware of GINA recommendations for children. Inhaled drugs are also more expensive than oral medications and many asthmatics are still presently treated by symptom relief with oral bronchodilators [11-14]. Aminophylline is widely used in most of the African countries because it is very cheap. In the study by Ayuk et al. [8], up to 73.3% of doctors still prescribed aminophylline for asthma management. Furthermore the inhaled drugs including the corticosteroids are not included in the national essential drug lists in a number of African countries [9, 12]. The recommendation of leaving a patient on a current ICS prescription for about 3 months before stepping up or down was poorly practiced although 70% of the respondents were familiar with the GINA guidelines. This agrees with findings from a previous study by Umoh et al [15] in Calabar, Nigeria and may be due to poor knowledge of the specific contents of the guideline. When giving metered dose inhaled medication, the use of spacer device for maximal drug deposition in the lungs has been recommended [1]. This was practiced by about half of our respondents. The study Desalu and colleagues [9] in Nigeria found that only 20.6% of the University teaching hospitals had spacer devices available in the clinics. Even if one had knowledge to use spacers appropriately, paediatric residents may have been limited by unavailability of spacers in their training centers. Only about a third of the residents self-reported providing asthma action plan for their patients.

In a study in South Africa, only 11.2% of patients received any self-management plan [8]. Their reported lower rate could be because of the retrospective nature of their study design. It is important that other forms of allergy especially in children must be assessed for, in every child with asthma. Asthma control would also be better if concurrent allergic rhinitis in kept under control. This practice was however poor among our residents. Asthma is a chronic illness which even though has no cure but with proper management patients have good quality of life. It is therefore imperative that patients be followed-up even when symptoms are no longer apparent. Majority of the resident did engage in regular follow-up of their patients. Exposure in terms of residency period clearly showed that those who had more than three years in training attested to better familiarity with GINA guidelines. However 11 of the 20 major practice points assessed were poorly practiced irrespective of the years of graduation, time in residency training or exposure to asthma CME. Among those who had exposure to additional asthma training, most had this training more than 5 years ago. This may explain why it did not impact on their practice and thus further reinforces the need to engage residents in frequent additional exposure to asthma training. It is also important to note that having many guidelines but not critically monitoring whether the implementation is effective leaves a huge gap when concluding that asthma prevalence is on the rise. It may be that physicians who see asthma patients have not been following these guidelines.

## Conclusion

The findings of this survey shows gaps in implementing guideline recommendations and this may be responsible for outcome of asthma control. It also serves as a pointer to what may be obtainable globally. The study has shown that there is need for knowledge reinforcement through further trainings, strengthening of paediatric pulmonology sub-units, provision of appropriate tools and need for support staff such as nurse asthma counsellors. While recommending country-specific asthma guidelines, more studies that assess the practical use of guidelines in other parts of the world are encouraged. This will help address the specific gaps in asthma management as has been identified in ours.

## What is known about this topic

- Asthma prevalence has continued to increase globally and the control of asthma especially in children has continued to be a challenge;
- Non-adherence to guidelines for asthma management by practitioners is noted as a cause;
- The paediatric residents in training within the teaching hospitals form a major bulk of the work force involved in the management of Nigerian children.

## What this study adds

- It is now known that 11 of the 20 major practice points emphasized in GINA guidelines were poorly practiced by our study participants irrespective of the years of graduation, time in residency training or exposure to asthma CME;
- This study highlights that while maintaining global standards, there is need to have locally adapted asthma guidelines that meet country-specific needs based on availability of resources as this may lead to better asthma outcomes in children;
- This study shows that to help address the specific gaps in asthma management especially in children, studies that assess and monitor the practical use of guidelines are invaluable as they may provide information on asthma outcomes in various countries.

## Competing interests

The authors declare no competing interest.
